# Gold Film over SiO_2_ Nanospheres—New Thermally Resistant Substrates for Surface-Enhanced Raman Scattering (SERS) Spectroscopy

**DOI:** 10.3390/nano9101426

**Published:** 2019-10-09

**Authors:** Karel Kouba, Jan Proška, Marek Procházka

**Affiliations:** 1 Department of Physical Electronics, Faculty of Nuclear Sciences and Physical Engineering, Czech Technical University in Prague, Břehová 7, 115 19 Prague 1, Czech Republic; Karel.Kouba@fjfi.cvut.cz (K.K.);; 2 Institute of Physics, Faculty of Mathematics and Physics, Charles University, Ke Karlovu 5, 121 16 Prague 2, Czech Republic

**Keywords:** SERS, AuFON, reproducibility, SiO_2_, thermal resistance

## Abstract

Surface-enhanced Raman scattering (SERS) sensors are constructed from metallic plasmonic nanostructures providing high sensitivity and spectral reproducibility. In many cases, irradiation of the SERS substrate by the laser beam leads to an increase of the local temperature and consequently to thermal degradation of metallic nanostructure itself and/or adsorbed analyte. We report here a “bottom-up” technique to fabricate new thermally resistant gold “film over nanosphere” (FON) substrates for SERS. We elaborated the simple and straightforward method of preparation of homogeneously and closely packed monolayer of SiO_2_ nanoparticles (50 nm in diameter) and covered it by a thin (20 nm) layer of magnetron-sputtered gold. The spectral testing using biologically important molecules (methylene blue, cationic porphyrin, and fungicide 1-methyl-1H-benzimidazole-2-thiol) proved a sensitivity and reproducibility of our AuSiO_2_ substrates. The main advantage of such SERS-active substrates is high thermal stability and low intensity of background and signal of graphitic carbon.

## 1. Introduction

Surface-enhanced Raman scattering (SERS) is an extremely sensitive spectroscopic technique employed in a wide variety of (bio)analytical, medical, and sensing applications [1,2]. It profits from the surface-plasmon resonance effect leading to an enormous enhancement of Raman scattering for molecules adsorbed to metallic (typically gold or silver) nanostructures [3,4]. The sensitivity and reproducibility of SERS-active substrates is a major requirement in designing and developing routine SERS quantitative applications. In this case, SERS-active substrate should: (i) provide high sensitivity (SERS enhancement better than 10^5^), (ii) provide enough spot-to-spot and batch-to-batch spectral reproducibility (signal variation less than 20%), (iii) provide a long shelf life (over weeks), (iv) have Raman spectrum and background itself as low as possible, (v) be easy and inexpensive to prepare and use [5,6].

The sensitivity and reproducibility of SERS-active substrates are directly related to the morphology of the nanostructures (i.e., size, shape, and spacing). High reproducibility of SERS signal over the whole substrate can be guaranteed at solid regular periodic nanostructural surfaces. In the past years, a big advance has been made in preparation of metallic nanostructures with optimal shape and size [7,8,9,10]. A variety of “bottom-up” techniques includes nanoimprinting [11], templating [12], and nanosphere or nanocolloidal lithography [13,14,15,16]. The metal “film over nanospheres” (FON) surface is formed by microspheres (e.g., polystyrene—PS) self-assembled on a solid substrate and then were coated by sputtered metal [17,18,19,20]. The main advantage of this technique is a rapid, inexpensive, large-scale (~cm^2^) preparation of SERS-active substrates with good spectral reproducibility. AuFON substrates were used for quantitative SERS detection, e.g., glucose and biowarfare agents [21].

In our previous papers, we reported an application of AuFON substrate based on Polystyrene (PS) spheres (107 nm in diameter, 20 nm gold coating) [20,22]. We were able to quantify the amount of colorant azorubine in sweet drinks [22] as well as to detect several biologically important molecules such as porphyrins, nucleic acids, benzocaine, and alkaloids [20]. However, polymer surfaces are not efficient in heat transfer (i.e., heat dissipation). Laser irradiation of the SERS substrate can lead to an increase of the local temperature and causes the thermal degradation of metallic nanostructures and/or adsorbed analyte. Degradation of the metallic nanostructure causes a significant increase of the spectral background connected with photoluminescence of the metal [23]. Moreover, decomposition of the analyte results in a decrease of the intensity of spectral bands, and the appearance of graphitic carbon spectral features (two broad peaks around 1200–1700 cm^−1^) [24]. In the case of AuFON substrates, we found that the Raman spectrum of PS, a mainly strong peak at 1000 cm^−1^, can also interfere in SERS spectra of the studied analyte [20]. Therefore, there is a need for inexpensive and stable SERS-substrates which can easily dissipate heat. Such substrates are expected to provide a SERS signal using high laser powers.

In this paper, we focus on the preparation of substrates based on monolayer colloidal crystals consisting of spherical SiO₂ nanoparticles covered by a thin layer of magnetron sputtered gold. Recently, SiO₂ microparticles (145–425 nm in diameter) covered by silver or gold have been used for SERS detection of proteins [25], textile fiber dyes [26] and organic dyes [27]. We improved previous preparations and elaborated the simple and straightforward method to obtain homogeneously and closely packed monolayer of SiO_2_ particles. The difference is also in the size of SiO_2_ particles (50 nm in diameter) in our case. The main advantage of SERS-active substrates prepared in this way is high thermal stability and low intensity of background signal because of the absence of organic materials, which can be a source of unwanted spectral interference (as for substrates based on polymeric nanoparticles such as PS). 

## 2. Materials and Methods 

### 2.1. Materials

Methylene blue (MB), 5,10,15,20-tetrakis(1-methyl-4-pyridyl)porphyrin (TMPyP), 1-methyl-1H- benzimidazole-2-thiol, trimethylchlorsilane (TMCS), SiO_2_ beads (50 nm in diameter, dispersed in ethanol) were purchased from Sigma-Aldrich (Prague, Czech Republic). Polystyrene (PS) spheres (aqueous dispersions, 107 nm in diameter) were purchased from microParticles GmbH (Berlin, Germany). 

### 2.2. Preparation of Surface-Enhanced Raman Scattering (SERS)-Active Substrates

AuFON substrates based on PS spheres (AuPS): These substrates were prepared according to our previous paper [20]. Briefly, PS spheres were mixed with ethanol (1:1 v/v) and deposited on a water surface. Then, the self-assembled PS monolayer was transferred on a silicon wafer and left to dry at ambient temperature. Finally, the monolayer was magnetron-sputtered (20 nm of gold, Cressington 208HR high-resolution sputter coater).

**Figure 1:**
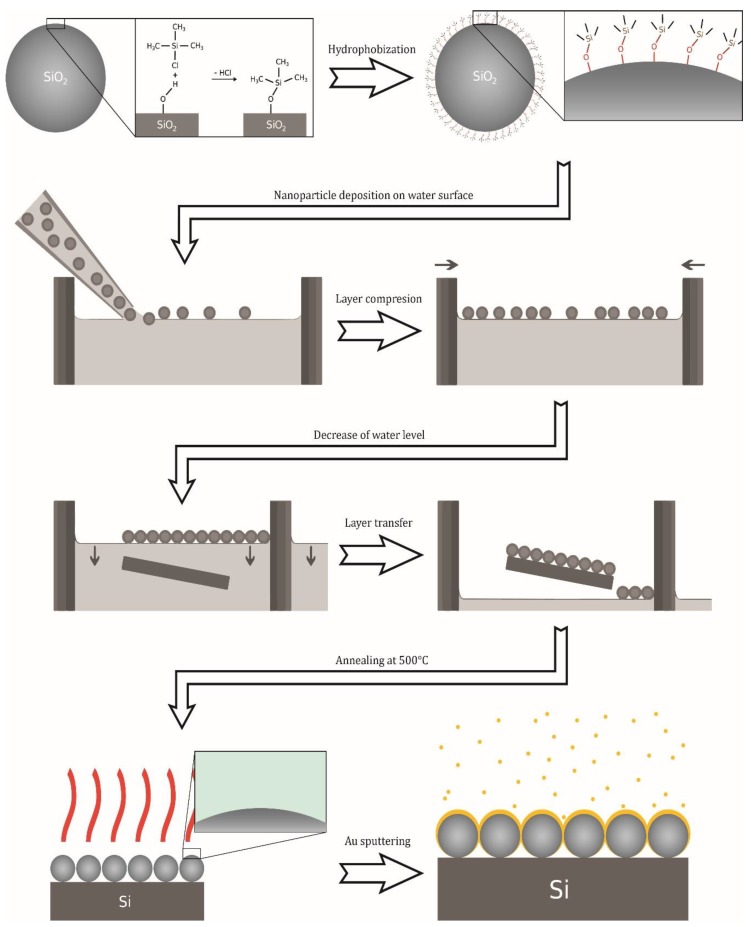
Schema of preparation procedure of AuSiO_2_ substrates.

AuFON substrates based on SiO_2_ (AuSiO_2_): Schema of the preparation procedure is depicted in Figure 1. Directed self-assembly of SiO₂ particles on the water surface is difficult due to the high density of silicon dioxide (the density of silica is higher than that of water and therefore it sinks when it gets below the water surface). To overcome this problem, we used the process of particle surface hydrophobization [28] modified with regard to the different starting material for the synthesis. The hydrophobization of the silica beads surfaces was carried out by the process of silylation using trimethylchlorosilane (TMCS). SiO_2_ beads were treated with 0.05% (v/v) TMCS ethanol solution. The pelleted beads were redispersed in 10 mL TMCS solution, sonicated for 10 min, left to stand for 16 h and shaken occasionally, followed by centrifugation at 10,000× g for 50 min, redispersed in 10 mL of absolute ethanol, centrifuged at 10,000× g for 50 min immediately, and a pellet was redispersed by sonication during 60 s in 3 mL of absolute ethanol. The final product was stored at 5 °C for further use. To produce a self-assembled layer, several μL of the SiO_2_ stock dispersion (diluted 1:1 with deionized water) were directed to the surface of water by micropipette.

**Figure 2:**
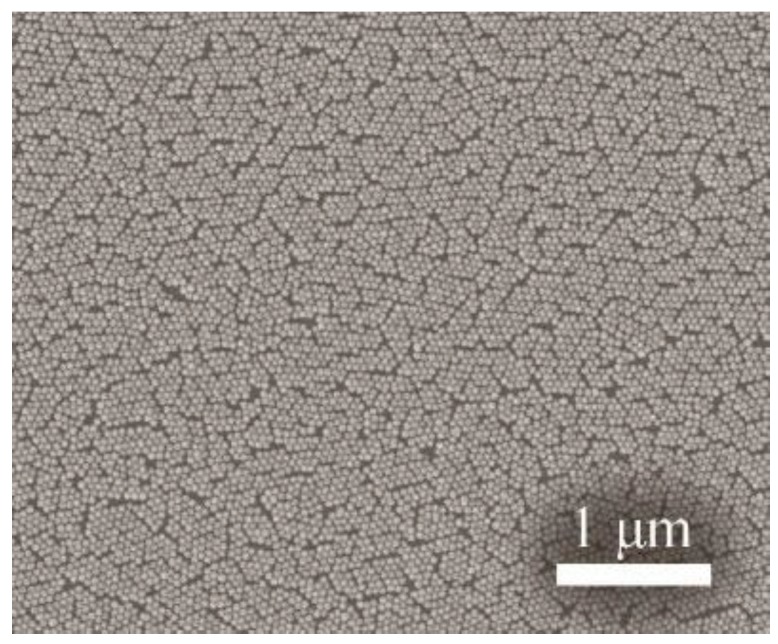
Scanning electron microscopy (SEM) image of a monolayer of SiO_2_ nanospheres (50 nm) covered by 20 nm of Au.

The formed layer was then mechanically compressed by using a movable wall of the vessel to ensure the closest packing of the nanoparticles. The compression was followed by lowering the water level until the entire layer was transferred onto the silicon wafer surface. The prepared monolayer was subsequently annealed at 500 °C for 2 h. Then, the self-assembled monolayer was coated with 20 nm of Au using a high-resolution sputter coater (Cressington 208HR). The AuSiO_2_ substrates were characterized by scanning electron microscopy (SEM, JEOL JSM-7500F). The typical SEM image of a monolayer of SiO_2_ nanospheres (50 nm) covered by 20 nm of Au is shown in Figure 2. It shows a very homogeneous packing density in the μm-scale.

### 2.3. SERS Experiments

All analytes were diluted in water except for 1-methyl-1H-benzimidazole-2-thiol which was diluted in methanol. The as-prepared SERS-active substrates were dipped in the solution of analyte (or water for bare substrates as true control) overnight and then dried in a nitrogen flow before Raman measurements. 

SERS spectra were acquired using the confocal Raman microspectrometer (LabRAM HR800, Horiba Jobin-Yvon) equipped with a nitrogen-cooled CCD detector. An objective 100× (NA = 0.9) focused a laser beam to a ~1.2 μm spot. A He-Ne laser (632.8 nm) was used as an excitation source. Laser power at the sample was adjusted using grey filters. Accumulation time was 1 × 60 s.

## 3. Results and Discussion

### 3.1. Comparison of Thermal Resistance of AuPS and AuSiO_2_ Substrates

**Figure 3:**
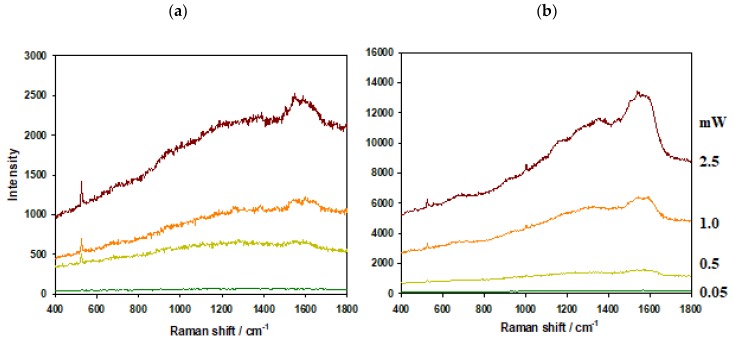
Bare substrates, (**a**) AuSiO_2_ and (**b**) AuPS. Laser power at the sample from bottom to top: 0.05, 0.5, 1, 2.5 mW.

**Figure 4:**
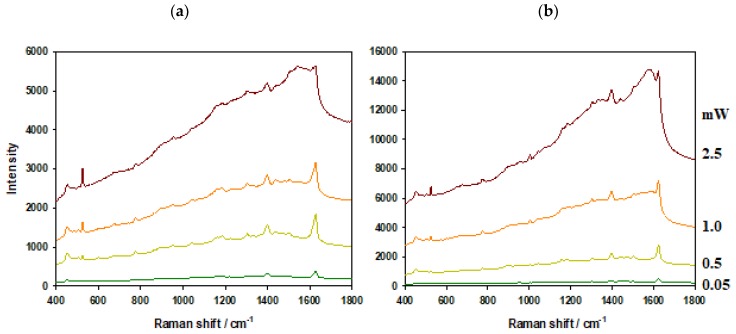
Surface-enhanced Raman scattering (SERS) of 0.1 μM methylene blue (soaking concentration), (**a**) AuSiO_2_ and (**b**) AuPS substrates. Laser power at the sample from bottom to top: 0.05, 0.5, 1, 2.5 mW.

To test the thermal resistance of AuPS and AuSiO_2_ substrates, we applied various excitation laser powers (0.05, 0.5, 1, 2.5 mW) to both bare substrates and the substrates with adsorbed MB. The same objective (100×, NA = 0.9) was used in all cases and focus was adjusted to the maximal signal. Figure 3 and Figure 4 show results for bare substrates and for substrates with adsorbed MB (0.1 μM soaking concentration), respectively. 

The background in the typical SERS spectrum is connected with photoluminescence of metal substrate [23]. In all spectra, the background is more pronounced for AuPS than for AuSiO_2_ substrates. In the case of bare substrates and high laser powers (1 and 2.5 mW), the background is about eight times higher for AuPS substrates in comparison to AuSiO_2_ ones. 

Moreover, two strong broad peaks of graphitic carbon around 1200–1700 cm^−1^ are visible in spectra obtained from AuPS substrates. In the case of adsorbed MB molecules and high laser powers (1 and 2.5 mW), the background and graphitic carbon bands almost overlap the spectrum of MB. The experiments with bare substrates proved that the graphitic carbon should be produced from the substrate itself (probably because of the decomposition of PS). 

Since AuSiO_2_ substrates show lower photoluminescence background and are more heat resistant than AuPS substrates, the higher laser powers can be used for SERS measurements.

### 3.2. SERS Testing of AuSiO_2_ Substrates

To test the sensitivity and reproducibility of our AuSiO_2_ substrates, we used various molecules and laser power 1 mW. 

**Figure 5:**
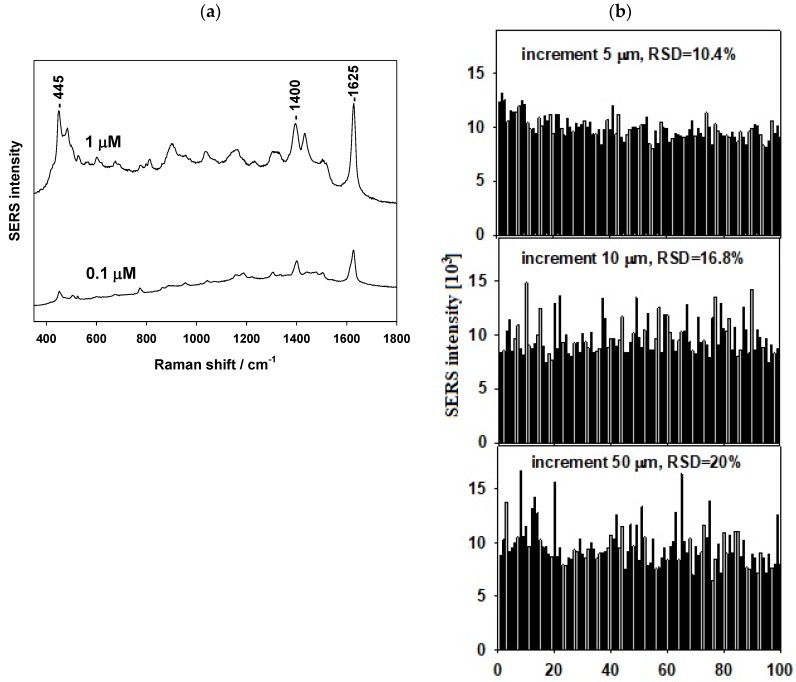
(**a**) SERS spectra of methylene blue and (**b**) spectral reproducibility (RSD—relative standard deviation of the signal) from 10 × 10 spectral maps of various increments.

First, the classical SERS probe (MB dye) was tested. Figure 5a shows SERS spectra of MB (1 and 0.1 μM soaking concentrations). Spectral reproducibility was tested by measuring spectral maps (10 × 10 spectral points) of MB (1 μM soaking concentration). Figure 5b shows the results for three such maps with increments of 5, 10 and 50 μm between the spectral points. Spectral reproducibility represented by the relative standard deviation (RSD) of the SERS signal was 10.4%, 16.8% and 20.0%, respectively. 

**Figure 6:**
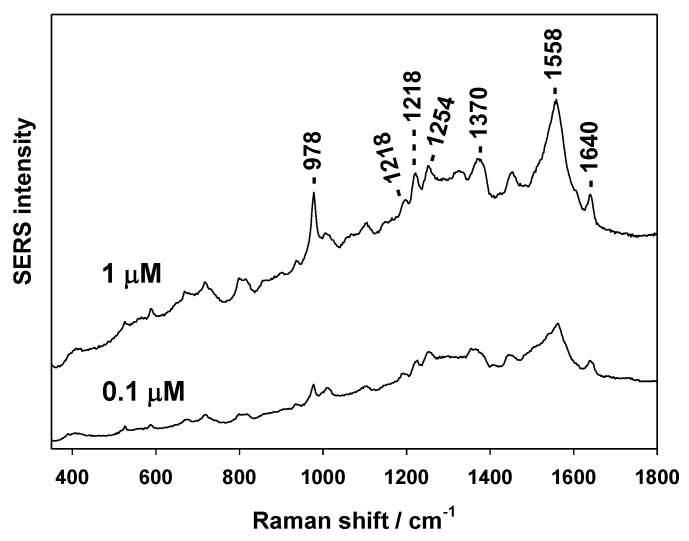
SERS spectra of 5,10,15,20-tetrakis (1-methyl-4-pyridyl)porphyrin (TMPyP).

The second tested molecule was cationic TMPyP porphyrin. It is a molecule providing a good SERS signal on Au substrates [20,29]. The SERS spectra of TMPyP (1 and 0.1 μM soaking concentrations) are shown in Figure 6. The SERS spectrum corresponds well to this recently obtained from AuFON on PS [20]. 

Similarly to the AuPS substrate, we determined the surface enhancement factor (SEF) of our AuSiO_2_ substrates using TMPyP. SEF is defined by the intensity ratio of SERS and normal Raman spectrum multiplied by the ratio of the average number of molecules providing both signals [4]. We used integral intensities of the TMPyP SERS spectrum obtained from the AuSiO_2_ substrate (1 × 10^−7^ M soaking concentration) when we suppose less than submonolayer coverage. In this case, the number of adsorbed TMPyP was estimated as ~10^14^ molecules cm^−2^. Normal Raman spectrum was measured under the same experimental conditions without the SERS substrate (1 × 10^−3^ M TMPyP concentration). The SEF or our AuSiO_2_ substrate for TMPyP was determined to be ~10^5^, similar to AuPS substrates [20]. 

**Figure 7:**
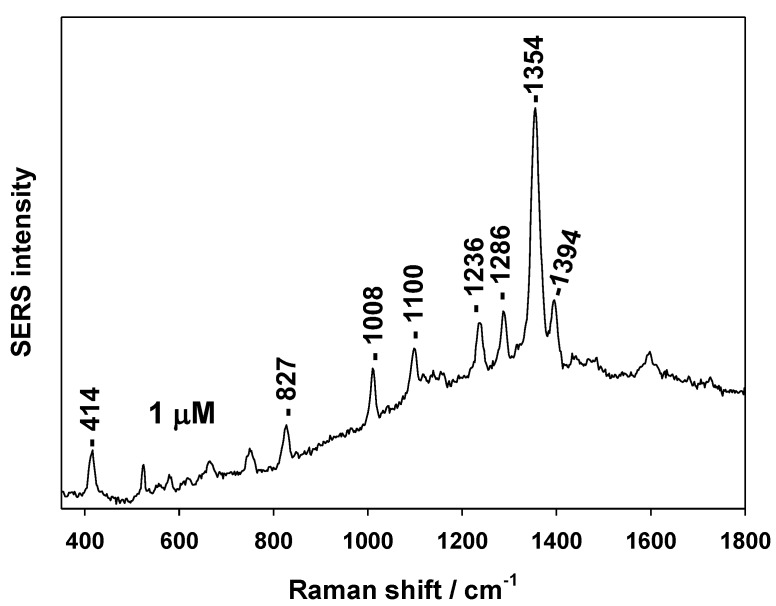
SERS spectrum of fungicide 1-methyl-1H-benzimidazole-2-thiol (1 μM soaking concentration).

Fungicides are chemical compounds used to kill parasitic fungi or their spores. They are used in agriculture but the fungicide residues can be detected in food for human consumption. Some fungicides are dangerous to human health. Thus, their detection and quantification in low concentration are necessary. To test the possibility of detect fungicides by SERS on AuSiO_2_ substrate we chose 1-methyl-1H-benzimidazole-2-thiol. It acts by binding to the fungal microtubules and impeding hyphal growth. Figure 7 shows the SERS spectrum of 1-methyl-1H-benzimidazole-2-thiol (1 μM soaking concentration).

## 4. Conclusions

We reported a “bottom-up” technique of preparation of thermally resistant gold “film over nanosphere” (FON) substrates based on SiO₂ nanoparticles (50 nm in diameter) covered by a thin (20 nm) layer of magnetron-sputtered gold. We elaborated the simple and straightforward method of preparation of homogeneously and closely packed monolayer of SiO_2_ nanoparticles. Sensitivity and reproducibility of AuSiO₂ substrates were tested using various molecular probes: MB dye, TMPyP porphyrin, and fungicide 1-methyl-1H-benzimidazole-2-thiol. The surface enhancement factor (SEF) for TMPyP was estimated to be at minimum 10^5^, similar to AuFON substrates based on PS spheres. RSD of MB SERS signal was less than 20%, which confirms the good spectral reproducibility of AuSiO₂ substrates. Our results clearly prove that AuSiO_2_ substrates have lower photoluminescence background and are more heat resistant to high laser power than AuPS substrates and therefore higher laser powers can be used for SERS measurements in the former case.
